# Mineral and Anthropogenic Indicator Inorganics in Urban Stormwater and Snowmelt Runoff: Sources and Mobility Patterns

**DOI:** 10.1007/s11270-017-3438-x

**Published:** 2017-07-05

**Authors:** H. Galfi, H. Österlund, J. Marsalek, M. Viklander

**Affiliations:** Department of Civil, Environmental and Natural Resources Engineering, Lulea University of Technology, S-971 87 Lulea, Sweden

**Keywords:** Snowmelt, Stormwater, Trace metal

## Abstract

Inorganic chemicals in urban stormwater and snowmelt runoff originate from catchment geology and anthropogenic activities. The occurrence, partitioning and mobility of six minerals and six trace metal (TM) indicators of anthropogenic activities were studied in stormwater, snowmelt and baseflow in four urban catchments, and the sampling of inorganics was supplemented by measurements of electrical conductivity (EC), pH and total suspended solids (TSSs). Minerals occurred at concentrations several orders of magnitude higher (1–10^2^ mg/L) than those of TMs (10^−2^–10^2^ μg/L) and reflected the composition of local groundwater seeping into sewers. Concentrations of Ca, K, Mg and Na were enhanced by baseflow contributions and followed closely the electrical conductivity. Al and Fe minerals occurred in insoluble forms, and their pollutographs were similar to those of TMs, whose concentrations mimicked, to some extent, the flux of TSS. The TMs with the highest and lowest particulate fractions were Cr&Pb and Cu&Zn, respectively. The concentrations of total TMs in snowmelt were two to four times higher than those in stormwater, and both sources likely exceeded some of the stormwater effluent limits (for Cd, Cu and Zn) proposed in Sweden. Where such concentrations depended on water hardness, the risk of toxicity might be reduced by elevated hardness of the monitored snowmelt and stormwater. Recognizing the *good* ecological status of the study area receiving water, Lake Storsjön, some protection against polluted runoff and snowmelt may be needed and could be achieved by implementing stormwater management measures controlling TSS and TMs.

## Introduction

Chemical characterization of urban stormwater has been reported extensively during the past 50 years, particularly with respect to stormwater impacts on receiving water quality (US EPA, 1983; Eriksson et al., 2007). The most frequently studied water quality parameters included total suspended solids (TSSs), nutrients (species of P and N), heavy metals, indicator bacteria (US EPA, 1983) and, less frequently, trace organics (Eriksson et al., 2007). Parallel to such efforts, though on a much smaller scale, urban snowmelt quality was also addressed in regions with seasonal snowpacks. Snowmelt pollution is associated with accumulation and release of such pollutants as TSS (Sillanpää and Koivusalo, 2013), chloride (Marsalek, 2003), heavy metals (Kuoppamaki et al., 2014; Valtanen et al., 2014; Moghadas et al., 2015) and trace organics (Björklund et al., 2011). While the effluents from conventional urban drainage mostly impact surface waters, the contemporary stormwater management, with emphasis on stormwater infiltration, creates new pathways of stormwater and snowmelt in the urban landscape and extends the associated environmental concerns also to stormwater storage facilities (Snodgrass et al., 2008), soils in infiltration zones (Kondo et al., 2016) and urban groundwater (Marsalek, 2003).

From a general perspective, stormwater runoff can be described as a process occurring across the urban landscape and hence reflecting both the local geology and anthropogenic activities (Frost et al., 2015; Taka et al., 2017). Taka et al. (2016) expressed a similar idea for metal concentrations in streams but used different descriptions of causative factors: the watershed soil type and land use. In this connection, two groups of inorganics are of interest: those of primarily mineral origin and those serving as indicators of anthropogenic activities, such as trace metals. Mineral inorganics are abundant in the lithosphere, vary regionally according to soil and bedrock composition and can be further enriched in natural waters by weathering and atmospheric deposition (Eriksson et al., 2007; Lidman et al., 2014). In boreal soils of high latitudes, the glacial bedrock consists of granite, silicate and carbonate minerals, and its chemical weathering contributes to the enrichment of Ba, Ca, Mg, Mn, Na, Si and Sr in soils and groundwater (Shotyk et al., 2010). Some contributions of mineral inorganics may also originate from attrition or wash-off of urban concrete surfaces, structures and sewers (Davies et al., 2010) and, most importantly, applications of road salts and traction agents (sand and grit) in winter road maintenance in regions with seasonal snow (Marsalek, 2003).

Trace metals (TMs) in stormwater and snowmelt originate from traffic (Kayhanian et al., 2012; Huber et al., 2016), wash-off or corrosion of buildings and structures (Sörme et al., 2001; Petrucci et al., 2014; Huber et al., 2016), atmospheric deposition (Gunawardena et al., 2013), crustal leaching (Joshi and Balasubramanian, 2010) and impurities in deicers and grit applied to roads in winter maintenance (Westerlund et al., 2003). Thus, while most of the TM burden is associated with anthropogenic activities, small contributions from mineral sources should be acknowledged as well, particularly in the case of Pb, Ni, Cu and Cr in peat soils (Cory et al., 2006; Lidman et al., 2014). Since TMs in stormwater/snowmelt may occur at the levels causing toxicity effects in receiving waters, they were identified as priority pollutants and potential indicators of anthropogenic inputs to natural waters (Eriksson et al., 2007; Singh et al., 2013). In the EU Water Framework Directive (WFD), the maximum allowable concentrations (MACs) were promulgated for five trace metals (Cd, Pb, Ni, Hg and Zn) to assess the environmental risks in natural waters and fisheries (2006/44/EC; European Commission, 2006).

There is a renewed interest in stormwater and snowmelt characterization with respect to mineral inorganics and major ions (Taka et al., 2017) for several reasons. Some (e.g. Ca) may increase pH and thereby reduce the risk of metal leaching from sediments (Salomons and Förstner, 1984) and provide the buffering of toxic effects (European Commission, 2013). Others (road salts) may cause toxicity effects (Marsalek, 2003), contribute to releases of chemicals from roadside environments (Amrhein et al., 1992) or (e.g. Na) adversely affect the properties of soils in green drainage infrastructure facilities with respect to retention of chemicals (Winston et al., 2016), soil structure and infiltration properties (Amrhein et al., 1992). Most of the research in this field focused so far on road salt effects, with Amrhein et al. (1992) reporting that Na^+^ cation replaced Ca^++^ and Mg^++^ cations in soils, and contributed to leaching out of metals (Cd, Cr, Cu, Fe, Pb and Ni) from soils and alteration of their structure. Similar concerns apply to stormwater discharged into (i) substrates of permeable pavements, from which salt-laden inflows may release particulates with adsorbed chemicals and temporarily reduce the facility environmental performance (Winston et al., 2016), and (ii) urban open spaces (green infrastructure), particularly with respect to stormwater/snowmelt salinity and sodium adsorption ratio (SAR) (Rahman et al., 2016). Finally, Kondo et al. (2016) examined the elemental composition of soils in green infrastructure facilities and noted that while the elements posing health risks occurred at the same concentrations upstream of and in these facilities, Cd, Hg and Pb concentrations exceeded the soil cleanup objectives, and calcium and iodine concentrations exceeded the reference levels.

Recognizing the growing interest in urban stormwater and snowmelt inorganic data in conventional or green drainage infrastructure (Kondo et al., 2016), potential impacts of inorganics on the receiving environment (Marsalek, 2003) and the relative dearth of such data in the literature (Taka et al., 2017), a study of mineral and anthropogenic origin inorganics in urban stormwater and snowmelt was conducted. Specific objectives included the elucidation of occurrences, dissolved and particulate partitioning and mobility of 12 inorganics in urban stormwater and snowmelt; six were primarily of mineral origin (Al, Ca, Fe, K, Mg and Na), and six were trace metals primarily associated with anthropogenic activities (Cd, Cr, Cu, Ni, Pb and Zn). These selections were made by examining the commonalities among three indicator lists found in the literature: the inorganic pollutants in the US EPA 129 Priority Pollutants list detected “frequently” in stormwater (US EPA, 1983), the inorganics in the list of stormwater priority pollutants identified by Eriksson et al. (2007) as “scientifically justifiable” for the assessment in the European WFD and the elements found by Frost et al. (2015) in stormwater pond sediments.

## Methods

### Sampling Sites

Samples of stormwater and snowmelt runoff, and baseflow (where occurring), were collected during selected time periods in four urban catchments located in the City of Östersund, Sweden. The city has a population of about 45,000 inh. (2010 data), is located in the middle part of Sweden (63.18° N, 14.64° E) and is drained by the traditional curb and gutter system discharging into a separate storm sewer system with multiple outlets. The built-up area of the city can be divided into a number of sewersheds with singular outlets discharging without treatment into Lake Storsjön, which is one of the largest lakes in Sweden (area = 464 km^2^, volume = 8020 km^3^) serving for drinking water supply, recreation and fishing. Lake Storsjön is classified as a water resource with a good ecological status, and as such, the lake water quality has to comply with the EU WFD regulations concerning raw drinking water and bathing water quality (2006/44/EC; European Commission, 2006).

To obtain information on characteristics of Östersund urban drainage effluents discharged into Lake Storsjön, four sewersheds with different land uses were selected as study sites serving for collection of samples at the last manholes immediately upstream of the sewershed outfalls. The study sewersheds were designated A–D, and their characteristics are summarized in Table 1.

**Table 1:** Characteristics of the Östersund sewersheds serving for sampling urban drainage effluents (stormwater, snowmelt and baseflow)

Sewershed (catchment)	Area (ha)	Imperviousness (%)	Baseflow in dry weather	Land use
A	19	21	No	Green areas (i.e. parkland and urban forest), with one residential street
B	21	50	Yes	Residential area with single-family homes on grassed lots
C	36	60	No	Central area with institutional buildings (university and municipal), parkland, roads and streets and residential housing
D	22	80	Yes	A hospital complex with surrounding parking lots and streets

The study area geology comprises the Jotnisk sandstone formation, overlaid by clayey till soils of glacial origin (Lundegårdh et al., 1984). Such formations and soils leached out major ions and alkali from sources of the Earth’s crust into the storm sewer baseflow as noted by Galfi et al. (2016).

Individual sewersheds are drained by dendritic storm sewer systems, with single outfalls from each sewershed. The drainage system comprises concrete sewers ranging in size from 0.3 to 0.6 m. Even though the sewers are in fairly good conditions, two of the studied sewersheds, B and D, experience ingress of groundwater in the form of continuous baseflows, which were also sampled to evaluate their effects on the composition of sampled stormwater and snowmelt.

### Field Sampling Procedures

Stormwater samples were collected in all the four study sewersheds (A–D) simultaneously to obtain samples from the same events. A total of six rain-driven events and six snowmelt events were sampled between September 2012 and June 2013. The rain-driven runoff events occurred and were sampled during the warmer part of the year and were associated with rainfalls ranging from 2.8 to 11.2 mm, preceded by dry periods varying from 3 to 14 days, allowing accumulation of pollutants. The snowmelt events occurred in late winter/early spring and were characterized by dry weather. Baseflows in sewersheds B and D were sampled on nine occasions in each sewershed during dry weather, between rain and snowmelt periods. Altogether, about 270 samples of stormwater, snowmelt and baseflow were collected during the study period and submitted for analysis of selected inorganics.

During individual sampling events of various durations, 4 to 15 grab samples were collected by dipping pre-rinsed 2-L polypropylene sample bottles into the sewer flow at the mid-section of the sewer. Discrete grab samples were collected proportionally to a predefined flow volume, using flow rates measured by an area-velocity flow meter (type ISCO 2150) installed in the storm sewer a short distance upstream of the sampling point. The frequency of sample withdrawal varied from catchment to catchment, with consecutive samples withdrawn after 1 to 40 m^3^ of water passed through the site, depending on the runoff intensity. After withdrawal, the samples were placed in coolers and submitted for analysis.

### Selection of Quality Constituents and Analytical Methods

The selection of water quality constituents to be studied was based on the study objectives aiming to elucidate differences between the two types of inorganics in urban drainage effluents: (i) inorganics of mostly mineral origin, affected by local soils and geology (Frost et al., 2015), and (ii) inorganics serving as indicators of anthropogenic influences (Eriksson et al., 2007). In studies of Frost et al. (2015), a set of 20 inorganics in sediments of stormwater management ponds included Al, Ca, Fe, K, Mn, Mg and Na in the group of minerals. This selection was adopted here with one exception, omitting Mn, which was found in the studied samples at low concentrations (<0.2 mg/L). The remaining six elements occurred at concentrations 6–123 mg/L and, in terms of abundance in the Earth’s crust, were among the top 10. Six anthropogenic indicators relevant to the study area were selected (as detailed in Section 1): Cd, Cr, Cu, Ni, Pb and Zn, and are further referred to as TMs. For all of these selected indicators, the recommended maximum annual concentrations in stormwater discharges into Swedish receiving waters were published (Alm et al., 2010): Cd_tot_ = 0.45 μg/L, Cr_tot_ = 15 μg/L, Cu_tot_ = 30 μg/L, Ni_tot_ = 20 μg/L, Pb_tot_ = 10 μg/L and Zn_tot_ = 90 μg/L. Such guidelines were proposed for controlling environmental risks of TM release in stormwater discharges into Swedish receiving waters.

Inorganics analyses were supplemented by analyses of electrical conductivity (EC), pH and TSSs to assist in data assessment and interpretations.

All chemical analyses employed in this study were done by a SWEDAC-accredited commercial laboratory (ALS Scandinavia), following the prescribed analytical and QA/QC procedures. For the analysis of inorganics, a subsample of 100 mL was withdrawn in the field from the total sample (2 L) and kept at cool temperatures prior to analysis. Before submission to the laboratory, a 50-mL aliquot was pre-filtered (filter pore size 0.45 μm) and both non-filtered and pre-filtered subsamples were submitted for total and dissolved inorganic content analysis, respectively. The inorganics were determined by inductively coupled plasma (ICP) sector field mass spectrometry (SFMS; Element, Thermo Scientific) and ICP optical emission spectrometry (OES; Optima, PerkinElmer) according to slightly modified US EPA methods 200.8 and 200.7 (US EPA, 2001), respectively, with the reporting limits listed in Table 2. Total concentrations were determined after autoclave-assisted digestion in 1.3 M nitric acid.

**Table 2:** Reporting limits of concentrations according to ALS Scandinavia Laboratories AB

	Elements											
	Minerals (μg/L)						Trace metals (μg/L)					
	Al	Ca	Fe	K	Mg	Na	Cd	Cr	Cu	Ni	Pb	Zn
Total concentrations	10	200	10	400	200	500	0.05	0.9	1	0.6	0.5	4
Dissolved concentrations	0.2	100	0.4	400	90	100	0.002	0.01	0.1	0.05	0.01	0.2

The rest of the whole-water sample was analysed for physico-chemical properties in the laboratory of the Östersund municipality. The content of suspended solids was measured by the TSS standard method by extracting and filtering sample aliquots through pre-weighed glass fibre filters (Whatman GF/A filter) (SE-EN 872 2005). EC was determined in the laboratory by a W3 InoLab EC probe. Sample water temperatures were measured on-line in the field by a precision linear thermistor embedded in the area velocity flow meter (ISCO 2150).

### Computational Data Analysis

Minerals, TM and TSS concentrations sampled in stormwater and snowmelt runoff, and baseflow, were used to calculate flow-weighted event mean concentrations (EMCs) as

$$ \mathrm{EMC}=\frac{\sum_{i=1}^n{V}_i{C}_i}{\sum_{i=1}^n{V}_i} $$

where the numerator is equal to the total flux of the sampled constituent, the denominator is equal to the total volume (*V*) during the sampled portion of the runoff event and subscripts *i* and *n* denote the *i*th sample and the total number of samples, respectively.

Mean EC and baseflow mean concentrations (measured prior to each event in a single sample) were calculated as arithmetic means. TM concentrations below detection limits were considered in EMC calculations as one half of the reporting limit values.

The cluster analysis (CA), employing the distance measures, was used to assess the relationships among uncorrelated groups of parameters, using concentrations in individual samples. Such data were skewed and, therefore, were log-transformed before analysis. The cluster correlation analysis was used for addressing similar temporal behaviour among parameter groups, assuming that similar variation patterns indicate similar sources. Finally, the Pearson correlation analysis was applied to the log-transformed TMs and TSS data to assess the common pathways of these water quality constituents in stormwater and snowmelt runoff.

## Results and Discussion

The study results are presented under five subheadings addressing stormwater and snowmelt export patterns of minerals and TMs, correlation patterns among the constituents studied, pollutographs of minerals and TMs during discrete events, partitioning of TMs and correlations with TSS and an environmental assessment of the mineral inorganics and TMs studied.

### Stormwater and Snowmelt Export Patterns of Minerals and Trace Metals

The EMCs of minerals (Al, Fe, Ca, K, Mg, Na), TMs (Cd, Cr, Cu, Ni, Pb, Zn) and TSS and the EC readings are presented in Table 4. EMCs of mineral inorganics were generally several orders of magnitude higher than those of TMs, with the highest values measured for two major ions, Ca and Mg, particularly in the two catchments conveying baseflow (B = residential and D = hospital area) (Table 4). Thus, the stormwater composition was affected by highly ionized baseflow originating from groundwater, which may carry higher annual loads of minerals from catchments B and D than stormwater runoff, assuming that baseflow occurs continuously throughout the year.

Minerals and TM EMCs were significantly higher in snowmelt runoff, with ECs and TSS concentrations up to two and five times higher, respectively, compared to rain runoff. Higher mineral and TSS concentrations in snowmelt were attributed to the grit materials applied as traction agents in winter road maintenance. pH of stormwater runoff from rain and snowmelt varied between 6.9–8.4 and 6.2–8.6, respectively. Such values are within the limits recommended by the European Commission (EC) for fresh waters supporting fish life (EC, 2006).

When comparing concentrations of minerals and TMs in rain runoff, snowmelt and baseflow, the highest concentrations of dissolved minerals were observed in baseflow, followed by snowmelt and rain runoff, and this ranking was identical to that of respective EC measurements. Such a similarity in ranking is expected, because EC measures total dissolved solids (TDSs) in water. Two minerals occurring predominantly in insoluble forms, Al and Fe, exhibited transport patterns similar to those of trace metals.

Trace metals were transported mostly as a particulate load with TSS, and hence, their concentrations followed those of TSS. TSS in stormwater reflected the strength of sources (snowmelt > rain runoff >> baseflow), with transport capacities of these three flows being comparable. The measured major ion concentration ranges (Ca, K, Mg) during rainfall runoff (Table 3) were comparable to those reported by Taka et al. (2017) for three cold-climate low-to-intermediate imperviousness catchments in Helsinki, Finland. In the latter catchments, road salt is used in winter road maintenance and this was reflected by much higher Na concentrations, up to 1017.5 mg/L, than those observed in the study area (Na EMC min–max range 1.1–8.6 mg/L) without salt use.

**Table 3:** Descriptive statistics [arithmetic mean EMCs (mean), standard deviations (StDev) and minimum–maximum EMCs (min–max)] of minerals, TMs, TSS, pH and electrical conductivity for three sources of sewer flow (rainfall runoff, snowmelt and baseflow) in four study catchments (A–D), with the total number of samples per catchment and min–max numbers of samples for individual constituents

	EMC	Minerals						Trace metals						TSS (mg/L)	EC (μS/cm)	No. of samples
		Al (mg/L)	Ca (mg/L)	Fe (mg/L)	K (mg/L)	Mg (mg/L)	Na (mg/L)	Cd (μg/L)	Cr (μg/L)	Cu (μg/L)	Ni (μg/L)	Pb (μg/L)	Zn (μg/L)			
Rain																
A = green area	Mean(StDev)	2.3(±0.4)	13(±4)	2.7(±0.6)	2.4(±0.3)	1.7(±0.5)	2.2(±0.6)	0.05(±0.01)	4.5(±1.4)	15(±3)	5.2(±1.0)	3.4(±0.9)	128(±19)	80(±29)	91(±19)	42
	Min–max	1.9–2.9	8–19	1.8–3.5	2.1–2.7	1.1–2.4	1.3–2.8	0.04–0.06	2.4–6.4	13–20	3.5–6.3	2.3–4.2	98–144	36–118	70–121	6–13
B = residential	Mean(StDev)	1.4(±0.5)	36(±15)	2.1(±0.9)	3.3(±0.6)	9.0(±4.0)	5.0(±2.1)	0.16(±0.05)	2.6(±1.0)	11(±3)	3.0(±1.0)	3.0(±1.3)	128(±23)	60(±34)	270(±110)	39
	Min–max	0.4–1.8	24–60	0.6–3.3	2.6–4.0	5.5–16.0	2.9–8.6	0.10–0.22	0.7–3.5	6–13	1.2–3.9	0.9–5.0	91–148	13–99	173–448	5–8
C = mixed land use	Mean(StDev)	2.5(±0.6)	27(±11)	3.4(±1.5)	3.4(±1.2)	2.8(±1.1)	2.7(±2.1)	1.22(±1.67)	5.7(±2.2)	53(±18)	5.2(±2.5)	7.8(±3.6)	788(±605)^a^	141(±110)	135(±31)	28
	Min–max	1.7–3.1	15–41	1.7–5.0	2.0–4.9	1.7–3.8	1.1–5.7	0.22–3.72	2.9–7.5	33–75	2.3–7.5	4.6–12.2	299–1665	50–301	95–168	4–11
D = hospital area	Mean(StDev)	1.6(±0.4)	41(±12)	3.4(±2.5)	3.1(±0.8)	6.5(±3.5)	3.0(±1.0)	0.06(±0.03)	3.3(±1.2)	36(±19)	4.8(±1.8)	3.0(±1.3)	88(±25)	69(±20)	236(±81)	32
	Min–max	1.0–1.9	27–58	1.5–7.7	2.3–4.2	3.6–11.3	2.0–4.5	0.03–0.11	1.7–4.6	22–69	3.1–7.7	1.4–4.8	47–114	38–87	143–335	4–9
Snowmelt																
A = green area	Mean(StDev)	6.4(±3.3)	27(±5)	8.8(±5.6)	5.2(±1.5)	4.9(±1.7)	9.0(±9.4)	0.05(±0.02)	11.2(±7.3)	25(±15)	12.8(±7.6)	6.9(±3.6)	163(±83)	289(±307)	184(±53)	22
	Min–max	1.7–9.3	21–33	1.8–15.3	3.0–6.5	2.7–6.8	2.5–23.0	0.03–0.08	2.1–19.7	7–44	3.1–21.5	1.9–10.3	43–227	34–733	149–261	5–6
B = residential	Mean(StDev)	6.5(±5.0)	65(±9)	9.9(±7.6)	6.2(±1.2)	16.0(±3.1)	12.4(±3.9)	0.09(±0.05)	11.9(±7.8)	21(±13)	11.9(±7.8)	7.0(±5.1)	111(±81)	250(±191)	445(±80)	37
	Min–max	0.7–12.7	54–76	1.1–18.1	4.6–7.8	11.4–20.0	6.2–16.5	0.03–0.16	1.0–23.0	4–39	2.0–20.5	1.1–13.4	20–226	24–468	336–579	5–8
C = mixed and use	Mean(StDev)	19.6(±6.2)	65(±17)	32.1(±11.2)	9.1(±2.5)	13.2(±4.4)	20.2(±8.1)	0.29(±0.10)	34.3(±11.7)	76(±28)	36.5(±11.8)	27.0(±11.5)	515(±214)	908(±324)	239(±64)	24
	Min–max	14.4–28.4	48–88	22.8–48.3	7.3–12.8	9.7–19.7	14.5–32.2	0.18–0.39	24.8–51.1	46–111	29.0–54.1	18.0–43.8	291–773	654–1381	188–324	4–10
D = hospital area	Mean(StDev)	12.4(±6.9)	123(±68)	20.8(±12.9)	8.4(±3.4)	14.2(±8.9)	8.9(±5.2)	0.14(±0.10)	23.6(±14.2)	56(±34)	25.4(±15.3)	17.1(±13.0)	202(±126)	604(±442)	349(±157)	23
	Min–max	5.8–18.6	48–192	8.8–32.0	4.6–11.7	5.5–22.3	2.8–15.1	0.05–0.26	9.9–37.7	20–89	11.1–40.8	5.4–34.2	87–318	227–1224	207–527	4–8
Baseflow																
B = residential		0.03(±0.03)	106(±8)	0.2(±0.1)	4.4(±0.4)	29(±2)	16(±2)	0.06(±0.01)	<0.9	1.2(±7.2)	0.8(±0.2)	<0.5	7(±6)	3.1(±2.9)	733(±36)	11
D = hospital area		0.07(±0.09)	134(±34)	0.8(±0.5)	4.9(±1.3)	29(±8)	11(±3)	<0.05	<0.9	7.2(±4.5)	1.4(±0.4)	<0.5	9(±4)	4.7(±4.5)	758(±308)	9

^a^The highest Zn concentration (five times higher than the rest of the samples) was 4920 μg/L at the beginning of the storm event of June 3, 2013; without this value, the mean would drop to 635 μg/L

The higher ion concentrations in snowmelt were explained by smaller volumes of snowmelt, compared to rainfall runoff events, and high supplies of grit applied in the studied catchments. Furthermore, in catchments B and D, baseflow originating from the ingress of local groundwater (Galfi et al., 2016) enhanced mineral concentrations in sewer flow during snowmelt periods. The main source of TSS in snowmelt was the grit applied in large quantities to streets and roads in winter road maintenance, which is typical for northern cities avoiding a widespread use of road salts. 

The measured concentrations of TMs in baseflow (Table 3) were frequently below the reporting limits (Cr and Pb) presented in Table 3 and generally compared well to those reported in the literature for groundwaters and natural streams in Sweden (Ledin et al., 1989). The background levels reported for groundwater in crystalline rock formations were 0.006–0.1 μg/L for Cd, 0.3–4 μg/L for Cu, 0.02–0.3 μg/L for Pb and 2–40 μg/L for Zn (Ledin et al., 1989). The measured background concentrations of Cu were somewhat higher in the hospital catchment D (7.2 ± 4.5 μg/L). In natural streams in boreal forests, background concentration ranges were reported as 0.5–1.8 μg/L for Cu and 0.4–2.1 μg/L for Ni (Lidman et al., 2014).

TMs and TSS concentrations were highest during snowmelt runoff and compared well to those reported in the literature for snowmelt runoff from catchments with similar degrees of development. The ranges of concentrations of Cr, Cu, Ni, Pb and Zn were comparable to those reported by Valtanen et al. (2014) for two catchments (imperviousness of 19 and 62%) in Lahti, Finland, for the cold season, though the Zn data from Östersund were more skewed towards high values. Comparably high Cu concentrations and even higher EC values (1200 mS/cm) were reported by Andradottir and Vollertsen (2014) for snowmelt in less-developed suburban catchments with road salting in Iceland, with maximum concentrations of 32 μg/L of Cu and 121 μg/L of Zn. Some of the measured mean TM concentrations in snowmelt in the studied catchments (including the residential catchment B) exceeded those reported for stormwater in the literature (Westerlund et al., 2003; Gasperi et al., 2014). For example, the mean concentrations of Cu and Zn in residential stormwater in France and the USA ranged between 15–38 and 126–212 μg/L, respectively (Gasperi et al., 2014; US EPA, 1983). In terms of average concentrations, the study area rain runoff and snowmelt possessed Cd, Cu and Pb concentrations comparable to those in road runoff data analysed by Huber et al. (2016) for roads with low traffic intensities (average annual daily traffic (AADT) <5000), but higher Zn concentrations (i.e. 515 and 788 μg/L in the central catchment C, vs. 212 μg/L reported by Huber et al. (2016). This would imply additional sources of Zn in catchment C, besides traffic.

### Examples of Discrete Event Pollutographs of Selected Minerals and TMs

**Fig. 1:**
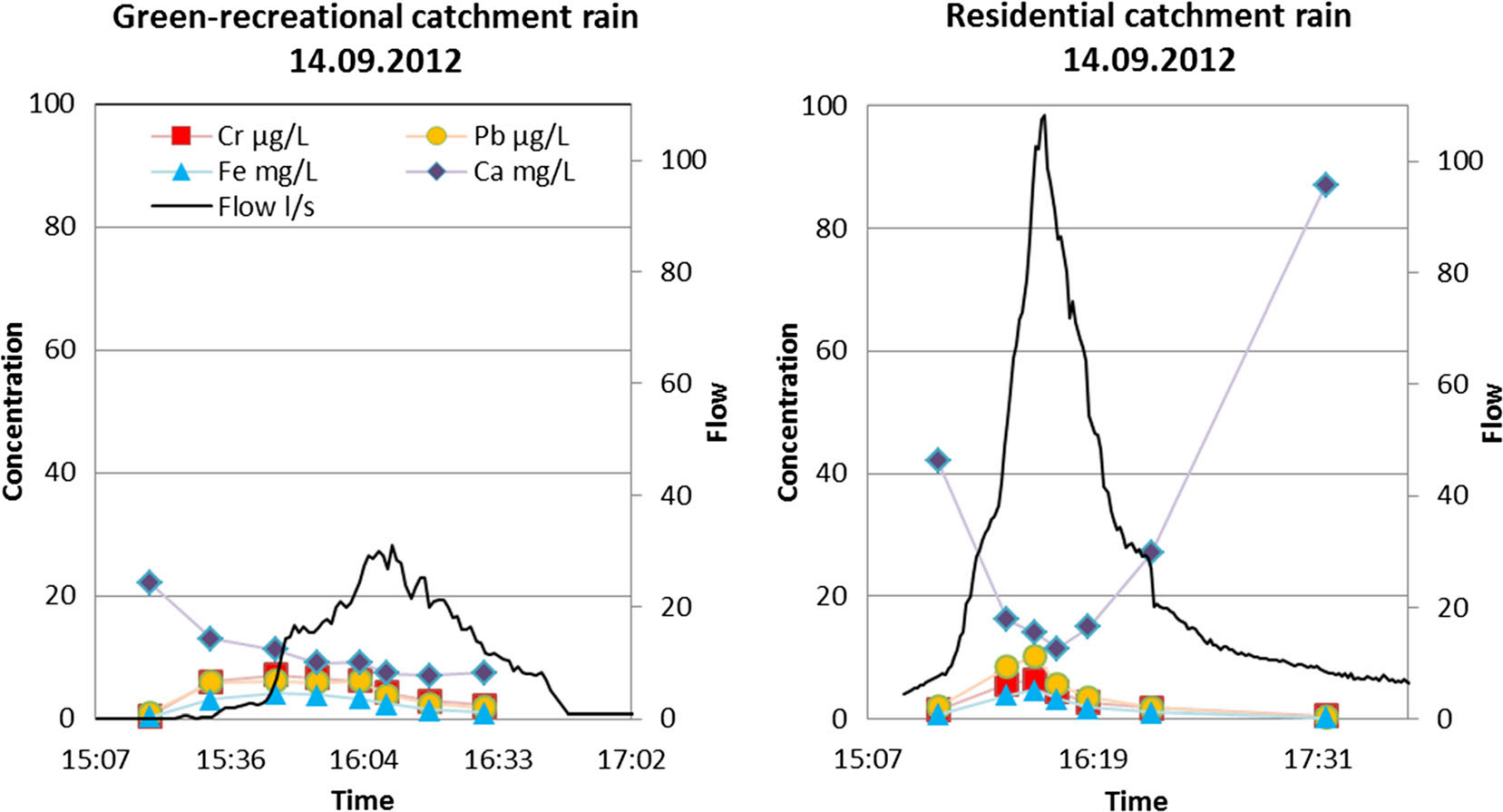
Hydrographs and pollutographs of four inorganics during rainfall/runoff events in two less-developed catchments studied (A and B) (Cr, Pb and Fe data represent particulate concentrations)

**Fig. 2:**
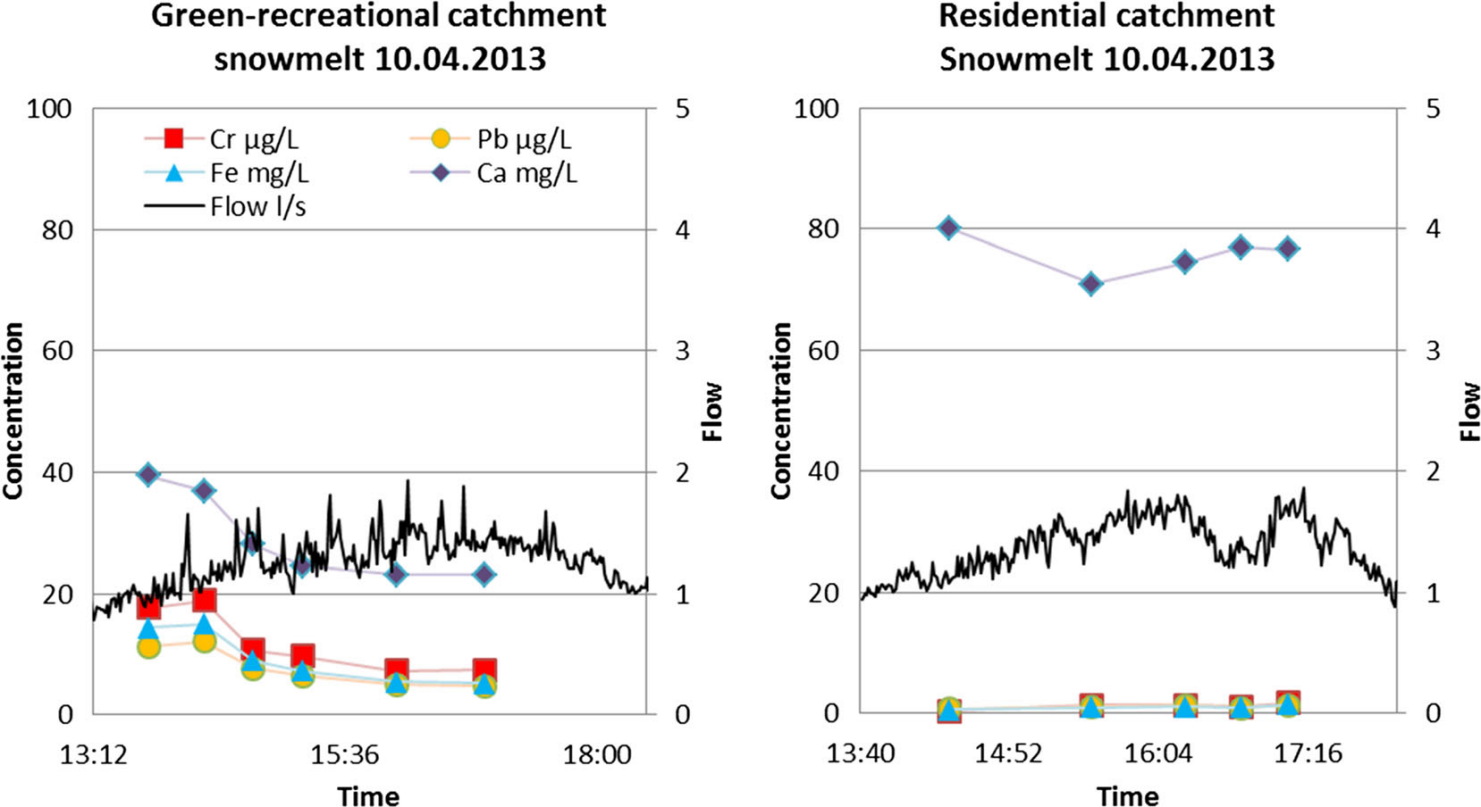
Hydrographs and pollutographs of four inorganics during snowmelt runoff events in two less-developed catchments studied (A and B) (Cr, Pb and Fe data represent particulate concentrations)

Pollutographs (i.e. graphs of concentration vs. time) of two minerals, Ca occurring prevalently in the dissolved fraction and Fe occurring mostly in the particulate fraction, and two TMs, Cr and Pb, occurring preferentially in the particulate fraction, are displayed in Figs. 1 and 2 to illustrate their transport pathways in discrete rain and snowmelt runoff events in two catchments with low-to-intermediate imperviousness (*i* = 21 and 50%, respectively). These events were selected so that they produced single-peak hydrographs during rainfall (September 14, 2012) and similar runoff volumes during snowmelt (April 10, 2013). Furthermore, Ca and Fe exhibited the highest concentrations among the minerals studied, in the dissolved and particulate fraction, respectively. Thus, the featured pollutographs demonstrate various flux patterns for typical examples of minerals and TMs studied. The dissolved constituent (Ca) behaved differently from minerals in the particulate fraction. Ca started in both catchments at relatively elevated concentrations (22–41 mg/L), which were quickly diluted as runoff flow increased. In catchment A, Ca flux followed a first flush pattern, and towards the end of the event, concentrations stabilized at low values. In the residential catchment B with baseflow, the concentrations followed a U shape; the initially high Ca concentrations (from baseflow) became diluted by stormwater but, towards the end of runoff, rose again as baseflow took over the control of Ca concentrations. Hence, in this case, the baseflow affected concentration variations during the event.

The remaining three elements, Fe, Cr and Pb, exhibited similar mobility patterns driven by transport of solids in sewers. At the start of hydrographs, during low flows, concentrations were rather low, increased with increasing flow (transporting more solids) and then declined with diminishing flow and supply of solids on the catchment surface. These patterns were similar in both catchments, in spite of differences in runoff flow discharges (the peak flow in the more impervious catchment B was about three times greater).

The snowmelt event (April 10, 2013) produced much smaller runoff flow rates (*Q*_max_ = 2 L/s) in both catchments, and Ca followed the same pattern as for rainfall runoff, with initial and end values influenced by baseflow, and the middle section reflecting dilution by snowmelt. Baseflow enrichment with Ca reflects supplies from sulphide-rich glacial soils (Lahermo, 1991) typical for the study area. The remaining parameters (Fe, Cr and Pb) started at low concentrations (low solid transport capacities), increased with increasing flows and gradually declined with depletion of particulate supplies. It was noted that peak concentrations exceeded those in rainfall runoff, perhaps because of high supplies of solids during snowmelt events. These findings agree with the earlier studies of heavy metals in snowmelt runoff in Sweden (Hallberg et al., 2007; Westerlund et al., 2003) and Germany (Helmreich et al., 2010). Concerning flow rates and inorganic concentrations, the more developed central catchments produced higher flows and TM concentrations, as also reported by Valtanen et al. (2014).

**Fig. 3:**
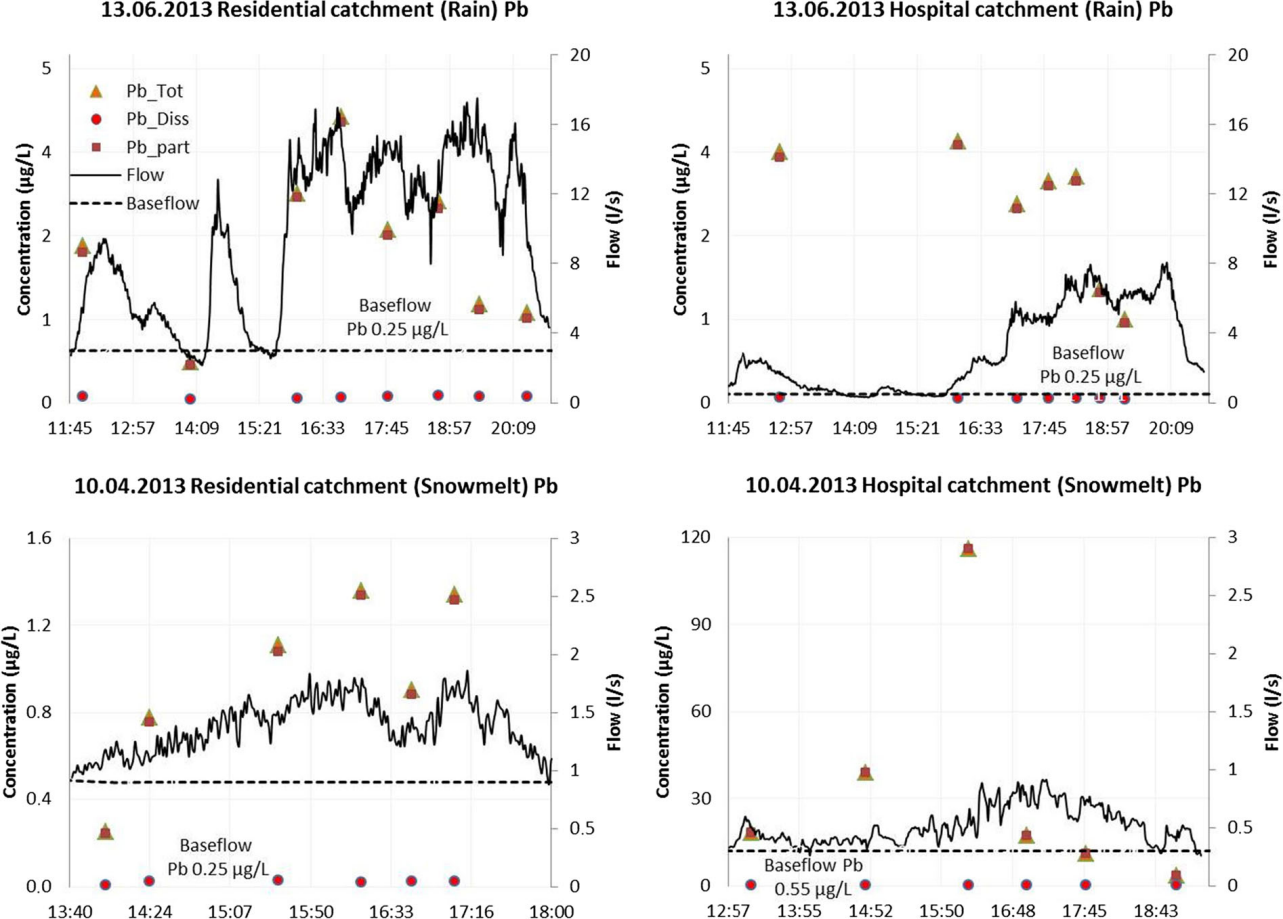
Total, dissolved and particulate Pb in the residential (B) and downtown hospital catchment (D) during rain runoff and snowmelt events

The pathways of total, dissolved and particulate Pb during rain and snowmelt events were exemplified by pollutographs for a rain event of June 13, 2013 and a snowmelt event of April 10, 2013, as shown in Fig. 3.

The pollutographs showed the following tendencies: (i) the graphs of total and particulate Pb (Pb_tot_ and Pb_part_) were almost identical, because of a negligible presence of Pb_diss_ (2–12%); (ii) both Pb_tot_ and Pb_part_ rain pollutographs displayed a weak first flush with concentration variations reflecting TSS transport, and exhaustion of TSS sources towards the end of the event; and (iii) in snowmelt, Pb concentrations were relatively small, because of low TSS transport capacity of the generated runoff flow (*Q* < 1 L/s).

### Correlation Patterns Among the Constituents Studied (Minerals, TMs, EC and TSS)

**Fig. 4:**
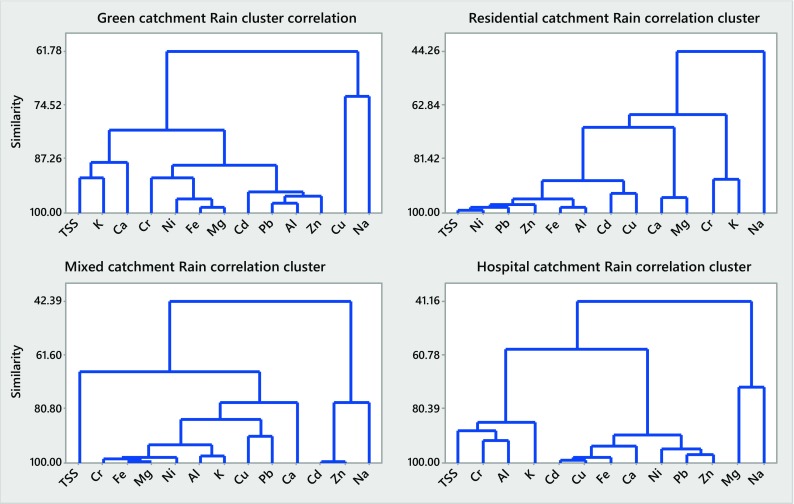
Correlation clusters for particulate concentrations in catchments A–D during rain-driven runoff

**Fig. 5:**
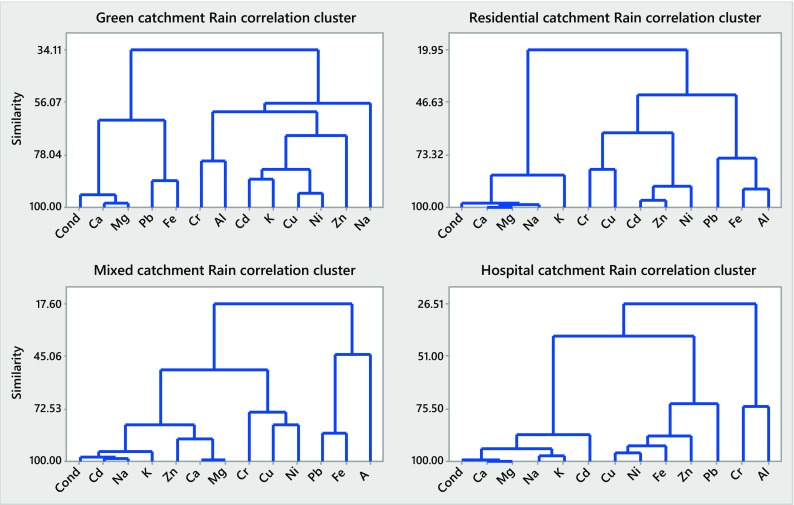
Correlation clusters for dissolved concentrations in catchments A–D during rain-driven runoff

**Fig. 6:**
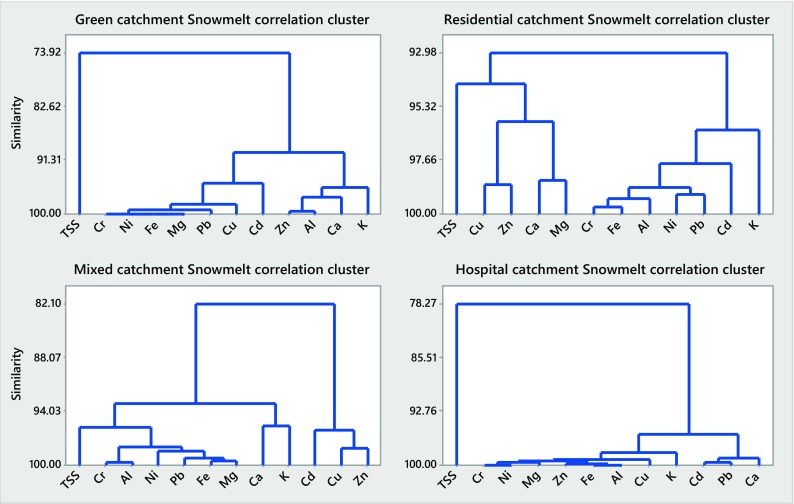
Correlation clusters for particulate concentrations in catchments A–D during snowmelt runoff

**Fig. 7:**
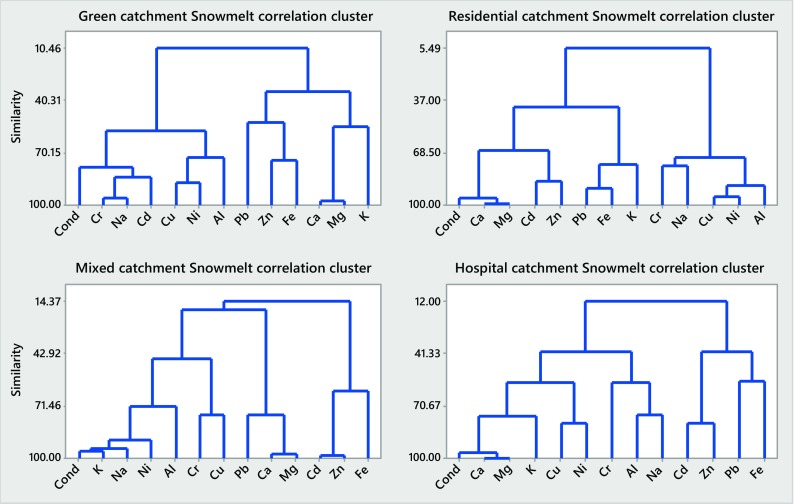
Correlation clusters for dissolved concentrations in catchments A–D during snowmelt runoff

Results of the cluster analysis of particulate and dissolved concentrations of the selected inorganics during rain runoff and snowmelt events in the four urban catchments studied are displayed in Figs. 4, 5, 6 and 7 and summarized in Table 4.

**Table 4:** Summary of cluster analysis (CA) results for rainfall and snowmelt runoff from the four urban catchments studied (A, B, C and D)

Concentration	CA similarities (%)									
	Minerals (Ca, K, Mg, Na) and electrical conductivity					Trace metals (Cd, Cr, Cu, Ni, Pb, Zn) and TSS				
	Catchment				Mean	Catchment				Mean
	A	B	C	D		A	B	C	D	
Rainfall runoff										
*C*_diss_	34	84	80	94	73					
*C*_part_						62	67	42	57	57
Snowmelt runoff										
*C*_diss_	10	5	18	41	18					
*C*_part_						74	93	82	78	82

Referring to Figs. 4, 5, 6 and 7 and Table 4, the CA findings can be summarized as follows: similarities determined by the CA for rainfall runoff and snowmelt runoff from the four catchments studied broadly vary and, therefore, with minor exceptions (particulate concentrations of minerals in snowmelt runoff), indicate trends in the data, rather than well-quantified findings. For minerals, high similarities were noted for dissolved concentrations in rain event runoff from the two catchments (B and D) with highly ionized baseflow (79 and 94%, respectively) (Fig. 5) and for particulate concentrations in snowmelt runoff (90–96%) from all four catchments (Fig. 6). The former observation confirms the strong influence of (groundwater) baseflow on catchment runoff with respect to concentrations of dissolved minerals; the latter one indicates the impact of grit applications on mineral concentrations in particulates. For TMs, the similarities among the six TMs studied and TSS were the greatest when examining their concentrations in particulates carried by snowmelt (74–93%, Fig. 6), but smaller among the concentrations in particulates carried by rainfall runoff (42–67%, Fig. 4). Hence, the presence of minerals and TMs in rainfall and snowmelt runoff is influenced by the presence of groundwater baseflow and high winter applications of road grit in cold climate.

### Total, Dissolved and Particulate TM Concentrations, and Correlations with TSS

**Fig. 8:**
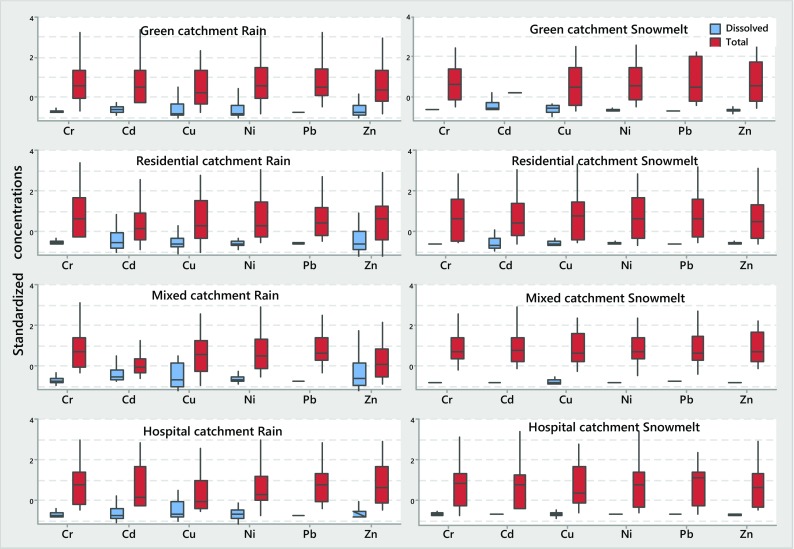
Box plots of normalized dissolved (*first column* in each pair) and total (*second column* in each pair) TM concentrations

TM concentrations were log-transformed, were normalized (i.e. individual EMCs were divided by the mean EMC from Table 3) to allow comparisons between sites and runoff sources (rain or snowmelt) and are presented in Fig. 8. The normalized log values calculated for total and dissolved concentrations ranged from −2 to 4 and indicated higher dissolved fractions during rain runoff, with the highest values attained in the central catchment (C) with mixed land use. A similar finding was reported by Valtanen et al. (2014), indicating that central catchments generally produced higher exports of pollutants than the suburban catchments.

Data on dissolved fractions of TMs studied, expressed as the percent of total TM burdens for the four catchments studied and rain or snowmelt runoff, are presented in Table 5.

**Table 5:** Ranges of mean dissolved fractions (%) for TMs in rain or snowmelt runoff from the catchments studied (A–D)

	Cd_diss_ (%)	Cr_diss_ (%)	Cu_diss_ (%)	Ni_diss_ (%)	Pb_diss_ (%)	Zn_diss_ (%)
Rain runoff						
A = green	15–40	1–18	30–60	24–49	2–8	25–56
B = residential	37–79	1–18	32–59	26–63	3–12	43–89
C = mixed	26–63	14–27	41–53	16–35	2–8	34–70
D = hospital	18–45	14–27	33–68	22–52	2–6	16–35
Snowmelt						
A = green	27–52	1–7	24–50	9–35	0–3	13–41
B = residential	14–100	1–13	14–40	6–50	0–7	5–81
C = mixed	2–16	2–3	12–20	3–5	0	1–15
D = hospital	6–17	5–11	17–30	9–20	0–2	4–16

In rain runoff (i.e. stormwater), among the six TMs studied, Cu and Zn exhibited the highest dissolved fractions, with the upper values exceeding 50% in at least three of the four studied catchments, and the relative magnitudes of dissolved fractions could be arranged, in a descending order, as follows:

$$ \mathrm{Cu}>\mathrm{Zn}>\mathrm{Cd}>\mathrm{Ni}>\mathrm{Cr}>\mathrm{Pb} $$

Such relatively high dissolved Cu and Zn concentrations agree with the previous findings of Gasperi et al. (2014) and Huber et al. (2016), reporting that more than 50 and 70% of these metal burdens were in dissolved fractions of residential and traffic-related runoff, respectively. These findings indicate that where removals of Cu and Zn would be contemplated, attention should be paid to processes targeting dissolved pollutants. At the other end of the list, Cr and Pb were predominantly associated with the particulate fraction (on average, 77–92 and 81–91%, respectively), which was in agreement with Gasperi et al.’s (2014) data (>80% of Cr and Pb in the particulate fraction) and Huber et al.’s (2016) analysis of traffic area runoff data. No clear trends were observed for Cd and Ni, with dissolved fractions broadly varying (on average, 24–57% for Cd and 22–50% for Ni). The relatively low concentrations of total Cd might be associated with natural inputs and atmospheric deposition (Davies et al., 2010).

In snowmelt runoff, the dissolved TM fractions were about one half of those in stormwater and reflected the abundance of TSS in snowmelt, compared to rain runoff. The relative magnitudes of dissolved TM fractions in snowmelt could be arranged in the descending order as follows:

$$ \mathrm{Cd}>\mathrm{Cu}>\mathrm{Zn}>\mathrm{Ni}>\mathrm{Cr}>\mathrm{Pb} $$

Total concentrations of TMs were highly correlated to TSS during both rain and snowmelt events, with statistically significant correlation coefficients (*p* < 0.05) shown in Table 6. Most exceptions were observed for Zn found in high concentrations in all catchments, indicating weak correlations with TSS during rain in the two catchments without baseflow (A and C). This might be explained partly by the variety of Zn sources in these two catchments including building facades, roofing materials and traffic (Mosley and Peake, 2001; Sörme et al., 2001; Petrucci et al., 2014), contributing to high concentrations of dissolved Zn, especially in catchment C.

**Table 6:** Pearson correlation coefficients between particulate concentrations of TMs and TSS during rain and snowmelt (statistically significant at *p* < *0.05*)

	Cr	Cd	Cu	Ni	Pb	Zn
Rain						
A = green	*0.7*	*0.7*	*0.6*	*0.7*	*0.8*	0.4
B = residential	0.3	*0.7*	*0.8*	*0.9*	*1.0*	*0.7*
C = mixed	*0.6*	0.3	0.4	*0.6*	*0.6*	0.3
D = hospital	*0.8*	*0.6*	*0.6*	*0.8*	*0.9*	*0.7*
Snowmelt						
A = green	*0.5*	*0.9*	*0.6*	*0.5*	0.4	*0.6*
B = residential	*1.0*	*0.7*	*0.8*	*0.9*	*0.8*	*0.8*
C = mixed	*0.9*	*0.7*	*0.9*	*0.9*	*0.9*	*0.7*
D = hospital	*0.8*	*0.6*	*0.8*	*0.8*	*0.7*	*0.8*

### Assessment of the Observed Mineral and TM Concentrations

Among the six mineral inorganics studied, none caused toxicity concerns about their occurrence in stormwater and snowmelt at the levels measured (Table 3). With respect to discharge of such waters into green infrastructure facilities, some guidance can be obtained from regulations for water reuse in irrigation of urban landscape, in which the main concern is about salinity of irrigation waters. This concern is typically described by EC of irrigation water, or TDS concentrations (derived from EC measurements), and/or by the SAR (Rahman et al., 2016):

$$ \mathrm{SAR}=\frac{{\mathrm{Na}}^{+}}{\sqrt{\frac{1}{2}\left({\mathrm{Ca}}^{2+}+{\mathrm{Mg}}^{2+}\right)}} $$

where Ca, Mg and Na concentrations are in milliequivalents per litre.

The Australian recycled water standards for irrigation allow the following recycled water characteristics: total salinity, measured as EC (dS/m) equal to 0.65–1.3 and SAR equal to 10–18 (Rahman et al., 2016). The maximum values of these characteristics observed in the study catchments were total salinity (EC) equal to 0.45 and 0.58 dS/m, in stormwater and snowmelt, respectively, and SAR equal to 0.15 and 0.6, for stormwater and snowmelt, respectively. These relatively low values of electrical conductivity and SAR follow from avoidance of road salting, and consequently, the stormwater and snowmelt from the study area could be safely discharged into green infrastructure facilities.

For the assessment of the measured TM concentration levels, a quick scan of available standards or guidelines was made and resulted in identifying three documents: EC environmental quality standards for priority pollutants (EC, 2013), the quality of fresh waters supporting fish life (EC, 2006) and TM limits for stormwater discharges into Swedish receiving waters proposed by Alm et al. (2010) in a report to the Swedish Water and Wastewater Agency. The first two represent standards for the ambient water quality and were not readily applicable to the data presented here. Thus, the third document was adopted, as it proposes maximum annual concentrations in stormwater discharged into the receiving waters for Cd, Cu, Ni, Pb and Zn (see Table 7). Such guidelines address potential cumulative effects of TMs, rather than acute effects in the form of acute toxicity. For this purpose, annual concentrations of TMs investigated in study catchments A–D were estimated, using the mean concentrations for rain runoff and snowmelt from Table 3 and apportioning them according to the estimates of annual volumes of rainfall runoff and snowmelt runoff. The results of such calculations are listed in Table 7 below; the values exceeding the proposed limits are presented in italics.

**Table 7:** Annual TM concentrations in stormwater runoff from catchments A–D and the proposed stormwater effluent limits (Alm et al., 2010)

Source/guideline	Total annual TM concentration (μg/L)				
	Cd	Cu	Ni	Pb	Zn
Catchment A	0.05	17	4	7	*135*
Catchment B	0.14	13	5	4	*124*
Catchment C	*1.0*	*35*	7	7	*452*
Catchment D	0.06	*37*	6	4	*96*
Proposed TM limits for stormwater (Alm et al., 2010)	0.45	30	20	10	90

Even with large uncertainties in the estimates of annual concentrations, there are a strong likelihood of TM limit exceedance for Zn in all the catchments studied and for Cd in catchment C and a much smaller likelihood of exceedance for Cu in catchments C and D. Decisions concerning the need to control the stormwater TM pollution in the urban area studied would require confirmation of TM impacts in the receiving waters and a more robust seasonal characterization of TMs in stormwater and snowmelt.

## Conclusions

The occurrence, mobility and partitioning of six abundant inorganics of primarily mineral origin (Al, Ca, Fe, K, Mg and Na) and six TM indicators of anthropogenic activities (Cd, Cr, Cu, Ni, Pb and Zn) were studied in stormwater and snowmelt runoff from four urban catchments in cold climate. In the context of study limitations, with respect to the number of events sampled and the number of samples, the following conclusions were drawn:

- The total mass of inorganics exported with runoff/snowmelt from the studied catchments was dominated by the inorganics of primarily mineral origin, which exceeded the mass export of TMs by several orders of magnitude, and reflected the composition of local groundwater.
- The presence of some mineral inorganics (particularly Ca and Mg) contributed to higher hardness of stormwater and snowmelt and thereby would provide some buffering of toxic effects of such waters. The measured mineral inorganics could be safely discharged into green infrastructure facilities, because of their low salinity (EC < 0.58 dS/m) and SAR (<0.6).
- The observed trace metal total concentrations in stormwater runoff were comparable to the literature values, except for higher Zn values indicating additional catchment-specific sources. Total TM concentrations in snowmelt were higher than those in rain runoff for most of the TMs studied, possibly because of long periods of accumulation of TMs in snowpacks and their storage in high TSS burdens resulting from grit applications in winter road maintenance.
- Particulate TM concentrations correlated well with TSS concentrations (in almost 83% of all cases, the correlation coefficient was >0.5). Thus, measurements of TSS could be used as surrogates for TMs and Al and Fe.
- The assessment of the observed TM concentrations against the proposed Swedish stormwater effluent limits (annual concentrations of five TMs) indicated the risk of exceedance in all the catchments studied for Zn and for Cd in central catchment C, and potential risks of exceedances for Cu in catchments C and D.
- Recognizing the *good* ecological status of the study area receiving water, Lake Storsjön, some protection against polluted runoff and snowmelt from the study area, and other contributing sewersheds, may be needed and could be planned, after increasing the robustness of the dataset on stormwater and snowmelt characterization. Reduction of pollution discharges into Lake Storsjön would be achieved by implementing stormwater management measures suitable for controlling TSS and TMs.
